# Using Virtual Reality to Rehabilitate Neglect

**DOI:** 10.3233/BEN-2012-129006

**Published:** 2013

**Authors:** A. Sedda, N. A. Borghese, M. Ronchetti, R. Mainetti, F. Pasotti, G. Beretta, G. Bottini

**Affiliations:** ^1^Humanistic Studies DepartmentPsychology SectionUniversity of PaviaPaviaItaly; ^2^AIS LabComputer Science DepartmentUniversity of MilanMilanItaly; ^3^Cognitive Neuropsychology CenterNiguarda Ca’ Granda HospitalMilanItaly; ^4^Neurorehabilitation and Rehabilitative MedecineNiguarda Ca’ Granda HospitalMilanItaly

**Keywords:** Virtual reality, neglect rehabilitation, neuropsychological rehabilitation, neural plasticity, grasping

## Abstract

*Purpose:* Virtual Reality (VR) platforms gained a lot of attention in the rehabilitation field due to their ability to engage patients and the opportunity they offer to use real world scenarios. As neglect is characterized by an impairment in exploring space that greatly affects daily living, VR could be a powerful tool compared to classical paper and pencil tasks and computer training. Nevertheless, available platforms are costly and obstructive. Here we describe a low cost platform for neglect rehabilitation, that using consumer equipments allows the patient to train at home in an intensive fashion.

*Method:* We tested the platform on IB, a chronic neglect patient, who did not benefit from classical rehabilitation.

*Results:* Our results show that IB improved both in terms of neglect and attention. Importantly, these ameliorations lasted at a follow up evaluation 5 months after the last treatment session and generalized to everyday life activities.

*Conclusions:* VR platforms built using equipment technology and following theoretical principles on brain functioning may induce greater ameliorations in visuo-spatial deficits than classical paradigms possibly thanks to the real world scenarios in association with the “visual feedback” of the patient’s own body operating in the virtual environment.

## 1. Introduction

Stroke is a leading cause of disabilities worldwide, as it can result in changes that leave about 30% of patients functionally disabled even years after the brain injury [1]. One of the most common consequences of right hemisphere stroke is visuospatial neglect, a complex neurological syndrome in which the patient is not able to interact, perceive and imagine stimuli located in the contra-lesional side of space [2]. Neglect also negatively influences recovery of motor impairment which requires longer rehabilitation [3].

The classical rehabilitative approach of neglect frequently does not generalize in the everyday like [4]. Virtual Reality (VR) platforms may be a valid alternative to the common rehabilitation techniques, as virtual tasks involve multisensory on-line feedbacks in a realistic context, providing better results in term of generalization of treatment effects [4]. Here we present a low cost VR platform apt to be implemented in a program of an at home rehabilitation.

## 2. Methods

Our platform equipments can be easily implemented at home. The platform setup is shown in Fig. 1. The screen where the virtual scene for the exercises performance is projected is placed at 1.5 m distance from the patient. The exercises scores are recorded in a host PC. The silhouette of the patient is acquired by means of a Sony PS3 "EyeToy" camera (640 $\times$ 480 pixels, 30 frames per second fps) and pasted in a virtual scene representing a virtual environment for exercises. An external assistance is not needed as the patient performs the task using his unimpaired hand. Thus, to acquire the collision points, the degree of overlap between the pa-

ISSN 0953-4180/13/$27.50 © 2013 – IOS Press and the authors. All rights reserved

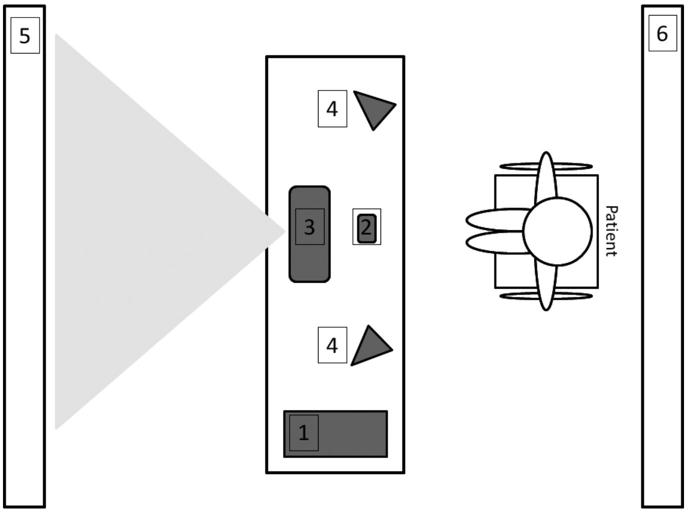

Fig. 1. System setup. 1) PC, 2) digital webcam with a resolution of 640 × 480 pixel at 60 Hz, 3) videoprojector, 4) stereo speakers, 5) white surface for projection (it can be substituted with a television screen), and 6) background white surface.

tient's hand and the object is measured when he touches a target.

The cognitive rehabilitation consists of a visual searching task [5]: targets are common objects, such as cans on kitchen shelves, randomly changing to maintain the attentional level. The level of difficulty of the task progressively increases due to the decrease of visual and auditory cues. Patients are asked to grasp the target object amongst distractors.

Our platform has been tested on patient IB (65 years old, 18 years of education) with a chronic neglect, following a right fronto-temporal intraparenchymal haemorrhagic lesion in 2009. He was treated in 2010. IB's family reported several difficulties in everyday life activities (such as reading and eating). IB was administered with an intense treatment for 4 weeks, 1 hour each day. Before starting IB underwent an extensive neuropsychological assessment, which confirmed the severe neglect with the exclusion of language impairment. IB underwent a post-treatment cognitive evaluation immediately after the rehabilitation and at 5 months. Written informed consent was obtained from IB for all the neuropsychological evaluations and rehabilitation sessions, and the treatment has been conducted in accordance with the Declaration of Helsinki.

## 3. Results

The neuropsychological tests (Attentional Matrices and the Mini Mental State Examination) demonstrated a significant amelioration of IB performance, also including a partial remission of the visuo-spatial impairment due to neglect as demonstrated by the line bisection test and by the Albert cancellation test. At the second follow up at 5 months, IB performance has proven to be stable. A self-reported amelioration of several everyday life activities (such as books and newspaper reading) has also been reported.

## 4. Conclusions

Our preliminary results suggest that VR platforms may help in the cognitive rehabilitation of complex neuropsychological syndromes such as unilateral neglect that are generally resistant to more classical treatment. In our view the general neuropsychological improvement beyond the visuo-spatial deficit due to neglect is encouraging suggesting that specific cognitive rehabilitation may modulate neural plasticity [6]. It is noteworthy that our treatment has been proven to be effective in a patient with a chronic, stabilized brain lesion. This evidence further supports that VR may favor plastic reorganization of the brain [7].

The novelty of our VR platform seems to ground on the inclusion of the full body view into real world scenarios, allowing patients to directly grasp objects.

## Declaration of funding

This work was partially supported by the Rewire project, funded by the EC under the FP7 framework, contract N. 287713.
